# Emotion regulation and psychological resilience sequentially mediate the association between training-related perceived stress and aggressive behavior among sport-major undergraduates

**DOI:** 10.3389/fpsyg.2026.1778128

**Published:** 2026-02-13

**Authors:** Yuanzheng Tan, Jianmin Ding, Guoquan Wang

**Affiliations:** Department of Physical Education, Guangzhou Xinhua University, Guangzhou, Guangdong, China

**Keywords:** aggressive, behavior, emotion, perceived, psychological resilience, regulation, serial mediation, sport-major undergraduates

## Abstract

**Background:**

Training-related perceived stress may be linked to aggressive behavior in university training environments, yet it remains unclear whether this association is transmitted through emotion regulation and psychological resilience and whether these processes operate sequentially.

**Methods:**

In a classroom-cluster convenience sample of sport-major undergraduates from a Chinese university (*N* = 711), participants completed standard measures with training-anchored instructions assessing perceived stress, emotion regulation, psychological resilience, and self-reported aggressive behavior. Measurement structure was evaluated using confirmatory factor analysis, and indirect and serial indirect effects were tested with bootstrapped mediation models (5,000 resamples; 95% percentile confidence intervals).

**Results:**

Higher training-related perceived stress was associated with higher aggressive behavior (total effect *B* = 0.183, 95% CI [0.123, 0.243]). Both emotion regulation and psychological resilience showed significant indirect effects, and a small but robust serial indirect effect through emotion regulation followed by psychological resilience was observed (serial indirect effect *B* = 0.004, 95% CI [0.001, 0.007]; total indirect effect *B* = 0.038, 95% CI [0.020, 0.058]). The direct association remained significant after accounting for the mediators (*B* = 0.145, 95% CI [0.084, 0.207]).

**Conclusion:**

Within training-anchored contexts, perceived stress is linked to aggressive behavior partly through constrained emotion regulation and reduced resilience, consistent with a “regulation-first, resource-next” sequence. These findings suggest a stepped prevention focus on reducing salient training stressors while strengthening emotion-regulation skills and resilience; longitudinal and multi-source studies are needed to test temporal ordering and causal mechanisms.

## 1 Introduction

**Figure 1:**
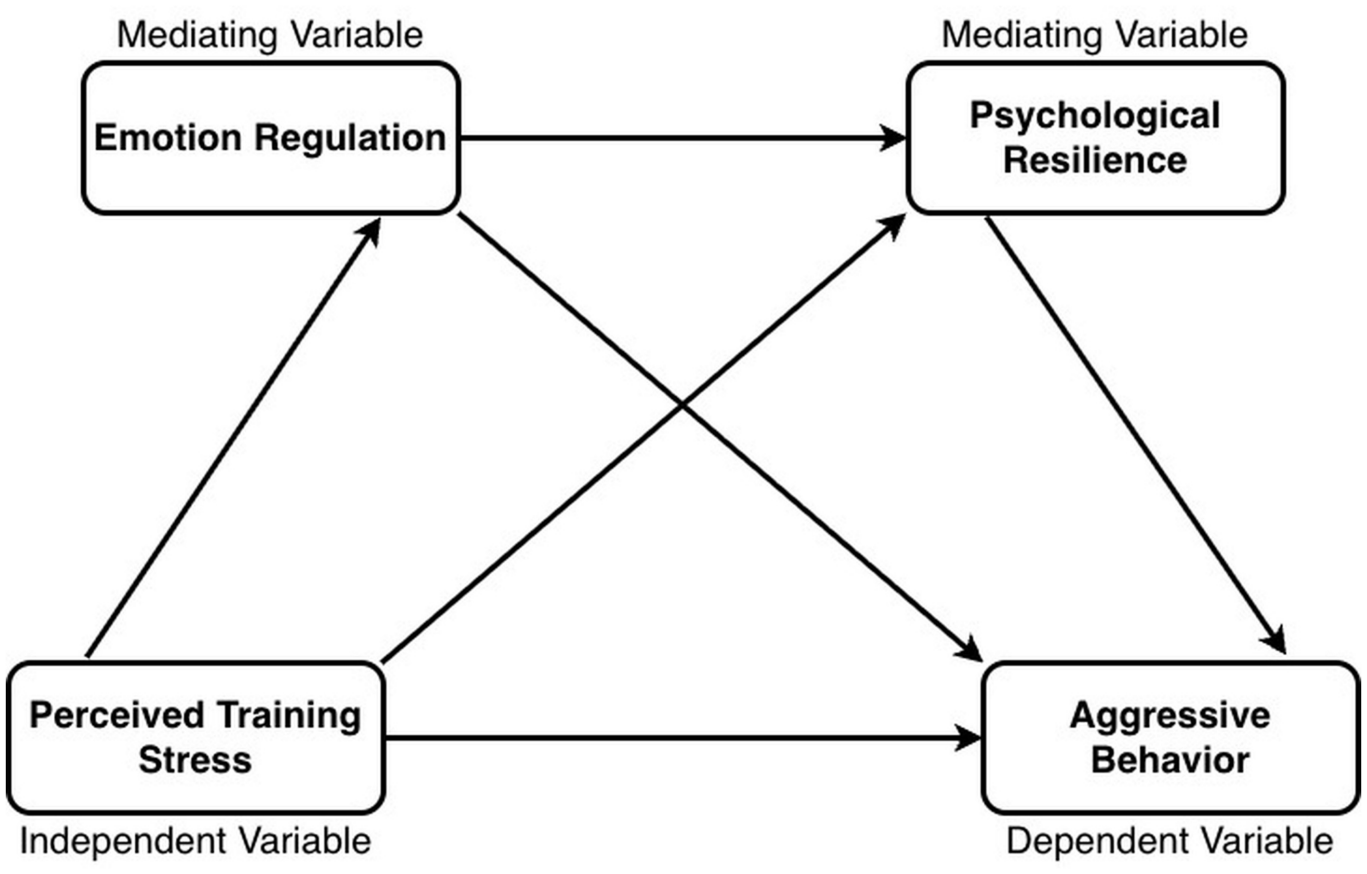
Hypothesized serial mediation model. Conceptual model linking training-related perceived stress (PTS) to aggressive behavior (AB) via emotion regulation (ER) and psychological resilience (PR), including the serial pathway PTS → ER → PR → AB, alongside the direct effect of PTS on AB. PTS, training-related perceived stress; ER, emotion regulation (higher scores indicate more reappraisal and less suppression); PR, psychological resilience (training-anchored perceived bounce-back capacity); AB, aggressive behavior.

**Figure 2:**
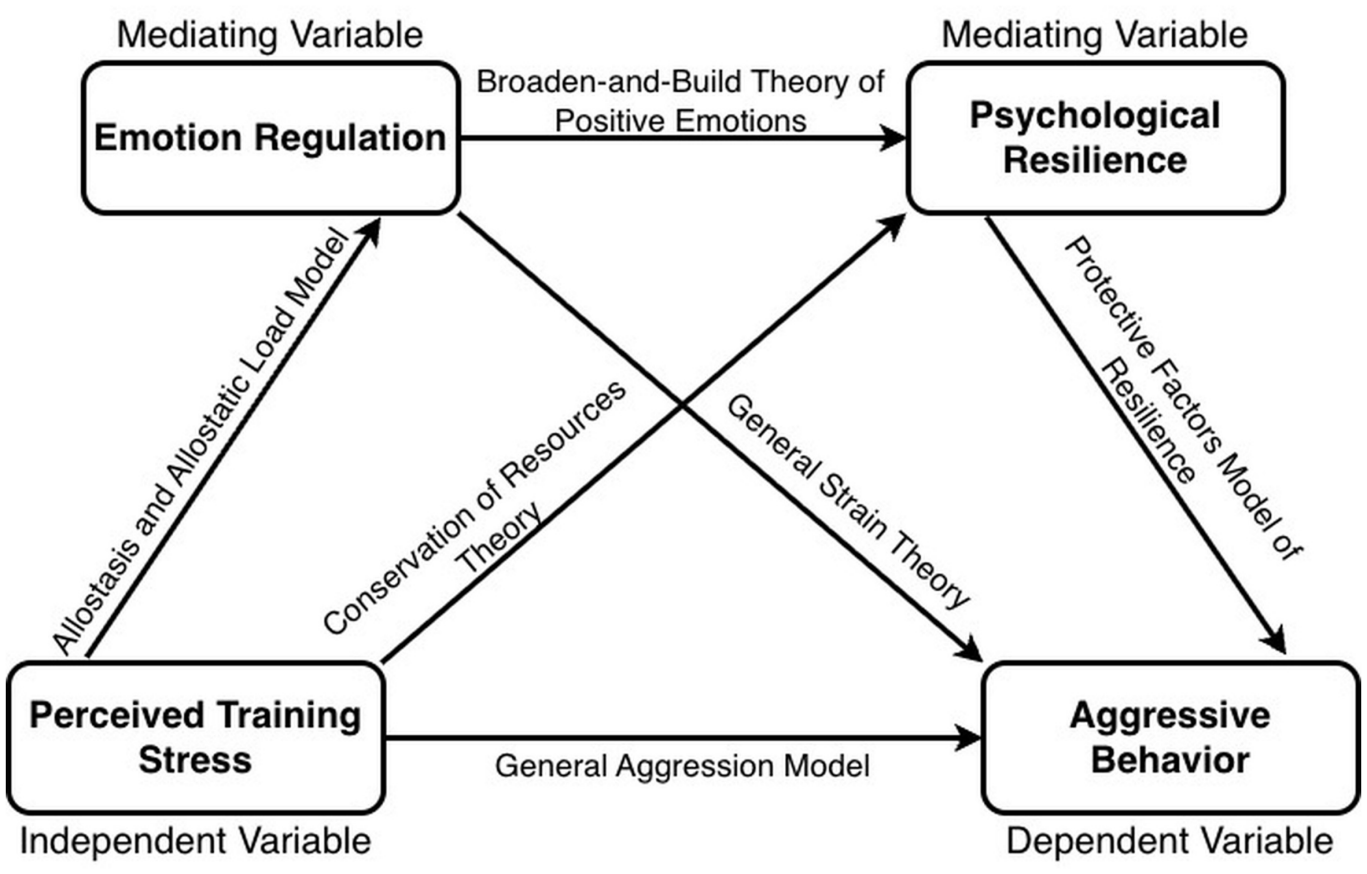
Theoretical framework supporting the hypothesized model. The figure summarizes the primary theoretical lenses used to motivate each proposed link in the serial mediation framework (e.g., PTS → AB; PTS → ER; PTS → PR; ER → PR; ER/PR → AB). PTS, training-related perceived stress; ER, emotion regulation (higher scores indicate more reappraisal and less suppression); PR, psychological resilience (training-anchored perceived bounce-back capacity); AB, aggressive behavior.

Mental health and behavioral risks among university students have become an increasing concern worldwide. Reports from UNESCO IESALC suggest that roughly half of students experience mental-health challenges during their studies; around 20% meet criteria for clinical depression, approximately 15% report suicidal ideation, and 2%–3% report suicide attempts, with campus closures and economic uncertainty adding further strain (UNESCO IESALC, 2024). In China, the National Health Commission launched the Action Plan for the Mental Health of Children and Adolescents (2019–2022) to raise core-knowledge awareness to 80% and strengthen school-based crisis response (National Health Commission of the People’s Republic of China, 2019). In 2024, the Ministry of Education initiated a “Year of Standardized Management” to intensify governance of school bullying and extreme behaviors (Ministry of Education of the People’s Republic of China, 2024). Sport-major undergraduates may face a distinctive configuration of stressors that combines academic demands with intensive training requirements, performance evaluation, coach–athlete interactions, roster competition, injury risk, and recovery constraints. In shared training environments, stress-related dysregulation may manifest not only as internal distress but also as externalizing behavioral risks (e.g., irritability, conflict escalation, and aggressive responses) that can undermine training climate and safety. Clarifying the psychological mechanisms linking training-anchored perceived stress to aggressive behavior is therefore relevant for prevention and governance within collegiate sport-training settings. The hypothesized serial mediation model is presented in Figure 1, and the integrated theoretical rationale is summarized in Figure 2.

Empirical work has linked perceived stress to aggressive behavior, yet most studies have focused on single or parallel mediators. As a result, integrative examinations that place emotion regulation and psychological resilience within a unified sequential process remain limited. Recent evidence suggests a moderate-to-strong positive correlation between emotion regulation and resilience (*r* ≈ 0.47; Stover et al., 2024). In adolescent and undergraduate samples, adverse life events (or stress) can translate into aggression via self-esteem and resilience (Yang et al., 2023), and emotion-processing pathways may likewise cascade to aggression (Zhou et al., 2024). However, under a training-related frame, direct evidence is scarce regarding whether stress is linked to aggression through a sequential “emotion regulation → psychological resilience” pathway, particularly among sport-major undergraduates. In sport and high-performance settings, psychological resilience has been examined as a key adaptation capacity shaped by salient stressors and protective factors relevant to training and competition (Sarkar and Fletcher, 2014). Sport resilience scholarship also highlights definitional and theoretical diversity, underscoring the importance of specifying how resilience is conceptualized and operationalized in a given study (Fletcher and Sarkar, 2013).

Against this backdrop, the present study focuses on sport-major undergraduates and—using a shared training-context anchor—integrates the General Aggression Model (GAM) and General Strain Theory (GST) with allostatic load and the Broaden-and-Build theory to propose and test a serial-mediation framework: training-related perceived stress (PTS) → emotion regulation (ER) → psychological resilience (PR) → aggressive behavior. The aims are to delineate the total and direct associations, estimate two independent indirect pathways via ER and PR, and examine the hypothesized “regulation-first, resource-next” sequence. Conceptually, the study seeks to provide chain-level evidence in a training ecology; practically, it informs a stepwise agenda of “load reduction-emotion-skills strengthening-resilience building” for governance of collegiate training environments. Table 1 provides an overview of the study aims, hypotheses, and analytic models.

**Table 1:** Overview of study aims, hypotheses, and analytic strategy.

Study objective	Hypothesis	Proposed relation (path)	Expected direction/pattern	Statistical test and estimation
Describe the overall association between training-related perceived stress and aggressive behavior	H1	PTS → AB	Positive association	Pearson correlation (two-tailed); total effect (c) estimated using OLS regression in PROCESS (Model 6) (unstandardized B with 95% CI)
Test the specific indirect effect via emotion regulation	H2	PTS → ER → AB	PTS negatively associated with ER; ER negatively associated with AB (overall positive indirect effect)	PROCESS model 6 (specific indirect effects); indirect effect tested using 5,000 bootstrap resamples (95% percentile CI)
Test the specific indirect effect via resilience capacity	H3	PTS → PR → AB	PTS negatively associated with PR; PR negatively associated with AB (overall positive indirect effect)	PROCESS Model 6 (specific indirect effects); indirect effect tested using 5,000 bootstrap resamples (95% percentile CI)
Test serial mediation (“regulation-first, resource-next”)	H4	PTS → ER → PR → AB	PTS negatively associated with ER; ER positively associated with PR; PR negatively associated with AB (overall positive serial indirect effect)	PROCESS Model 6 (serial mediation); serial indirect effect tested using 5,000 bootstrap resamples (95% percentile CI)

PTS, training-related perceived stress; ER, emotion regulation (higher scores indicate more reappraisal and less suppression); PR, psychological resilience (training-anchored perceived bounce-back capacity); AB, aggressive behavior.

### 1.1 Perceived training stress and aggressive behavior

The General Aggression Model (GAM) and General Strain Theory (GST) suggest that perceived training stress (PTS) can be conceptualized as both a situational input and a form of strain that increases aggressive responding through three channels—negative affect, physiological arousal, and hostile cognitions—while chronic strain may further erode self-control and reinforce hostile scripts (Slocum and Agnew, 2017; Allen et al., 2018). Biopsychological and experimental findings converge: reactive aggression is closely coupled with stress physiology, including HPA-axis activity and heart-rate reactivity (Romero-Martínez et al., 2022); undergraduates show higher aggression after social-evaluative stress, whereas familiarity and predictability attenuate this effect (Wu et al., 2021); and acute, uncontrollable stress is especially likely to trigger maladaptive/reactive aggression (Farkas et al., 2023). Taken together, training and competition environments—marked by high loads, public evaluation, and roster competition—are expected to show a positive PTS–aggressive behavior association.

Field and educational samples point in the same direction. In high-pressure occupations, perceived stress correlates positively with anger/irritability and aggression, and this association is weakened by problem-focused coping and positive cognitive reframing (Spaan et al., 2024). Among law-enforcement personnel, stress shows moderate associations with overall and affective forms of aggression, highlighting the sensitivity of the anger–hostility pathway (Christopher et al., 2024). Studies with adolescents and undergraduates likewise indicate that adverse life events raise aggression directly and indirectly via depleted psychological resources; regular exercise tends to covary with lower aggression, consistent with a “load reduction → less negative affect → reduced aggression” pathway (Yang et al., 2023; Liu et al., 2024). However, most prior work has not employed training-anchored stress measures nor targeted sport-major undergraduates. Based on this, Hypothesis 1 is proposed: Training-related perceived stress is associated with aggressive behavior.

### 1.2 Emotion regulation as a mediator

Allostatic load and the Process Model of Emotion Regulation posit that acute and chronic stress tax top-down control within prefrontal–limbic circuitry. This constrains antecedent-focused strategies such as cognitive reappraisal while privileging response-focused tactics (e.g., expressive suppression) and ruminative processing—an appraisal-control pathway consistent with the aggression-generation logic of the General Aggression Model and General Strain Theory (Gross, 1998; Shields et al., 2016). Meta-analytic work shows that stress reliably impairs working memory and cognitive flexibility and compromises complex inhibitory control (Shields et al., 2016). Moreover, the interplay between emotion dysregulation and inhibitory control predicts aggression and involves right prefrontal structures (Bounoua et al., 2022). The impact of stress on reappraisal is not uniform: effects vary by temporal window and individual differences—group means may be unchanged over short intervals; women with stronger sympathetic activation show greater decrements; and distraction during the delayed cortisol rise can partially offset costs (Langer et al., 2020; Langer et al., 2023; Sandner et al., 2021). In training-anchored contexts, periods characterized by heavy load, social-evaluative pressure, and uncertainty are therefore most likely to be accompanied by reduced regulatory resources and weaker inhibitory control.

Cross-context evidence links this regulatory pathway to aggression. In driving samples, emotion dysregulation significantly mediates the association between life stress and aggressive driving (Herrero-Fernández et al., 2024). In community samples, ER mediates the effects of sleep restriction on perceived stress and multiple facets of aggression (Demichelis et al., 2023). With respect to strategy quality, college students who rely more on reappraisal report lower aggression, whereas expressive suppression relates to higher aggression, with negative affect bridging these links (Gutiérrez-Cobo et al., 2023). Meta-analyses further indicate that reappraisal/acceptance correlate negatively with anger, while suppression/rumination/avoidance correlate positively; brief mindfulness interventions produce small-to-moderate reductions in anger and aggression (Pop et al., 2025; O'Dean et al., 2025). In sport ecologies, third-person self-distancing causally reduces athletes’ aggressive responses; maladaptive cognitive strategies predict competitive anger; life stress influences depletion via cognitive strategy use; and mindfulness relates to lower aggression indirectly through reduced emotion dysregulation (Michel-Kröhler et al., 2021; Behjame et al., 2021; Ma et al., 2025; Garofalo et al., 2020). Taken together, ER functions as a proximal, modifiable hub through which stress may translate into aggression, yet direct mediation tests using training-anchored stress measures among sport-major undergraduates remain scarce. Based on this, Hypothesis 2 is proposed: Emotion regulation mediates the relationship between training-related perceived stress and aggressive behavior.

### 1.3 Psychological resilience as a mediator

Conservation of Resources (COR) theory conceptualizes sustained or peak stress as a threat to—and depletion of—key psychological and social resources (Hobfoll et al., 2018). In the present study, psychological resilience (PR) is conceptualized as a resilience-related capacity/resource supporting recovery and behavioral self-control under strain (e.g., goal persistence, problem solving, and support mobilization), and is operationalized as training-anchored perceived “bounce-back” capacity over the past month. Consistent with sport psychology accounts, resilience is often described in terms of athletes’ ability to “bounce back” from adversity across training and competition contexts (Galli and Vealey, 2008). Grounded-theory work with Olympic champions further suggests that resilience under performance pressure is supported by protective factors that help athletes withstand and rebound from major stressors (Fletcher and Sarkar, 2012). Resilience has also been extensively examined in sport and high-performance settings as a key adaptation capacity under sustained training and competitive pressure. Positioning PR within a collegiate training ecology therefore strengthens the domain relevance of the proposed resource-based pathway. Consistent with these accounts, studies with Chinese undergraduates have shown that childhood adversity relates negatively to resilience and positively to aggression, with resilience significantly mediating the adversity-to-aggression pathway (Guo and Chen, 2015). In high-pressure healthcare settings, a stress → resilience → psychological distress chain has been documented, and workplace violence has indirectly worsened mental health via reduced resilience (Lara-Cabrera et al., 2021; García-Izquierdo et al., 2025). Resilience is inversely associated with aggression, anger, and hostility, and lower resilience has mediated the link from impulsivity to self-directed aggression (Naseem and Munaf, 2020; Ran et al., 2022). Within training-anchored contexts, prolonged or peak training load and competitive evaluation are likely to drain resources and constrain recovery, positioning resilience capacity as a key resource-based pathway linking stress to aggressive responding.

Evidence from educational and sport settings also indicates that resilience is malleable. Among female adolescent athletes, resilience subdimensions negatively predict aggression, and athletes tend to score higher on resilience than non-athlete peers (Nooripour et al., 2022). In other frontline high-stress roles, greater resilience relates to less outward anger expression (Jang et al., 2018). An eight-week resilience program has reduced anger expression in highly impulsive students, implying intervention leverage on aggression precursors (Sadri Damirchi et al., 2018). Beyond risk reduction, resilience has attenuated the erosive impact of negative cognitions on adaptation, underscoring its status as a protective resource (Yildirim, 2019). Nevertheless, few studies have explicitly tested resilience as a mediator when both the predictor (training-related perceived stress) and the outcome (aggressive behavior) are anchored in the training ecology of sport-major undergraduates. Based on this, Hypothesis 3 is proposed: Psychological resilience mediates the relationship between training-related perceived stress and aggressive behavior.

### 1.4 Serial mediation of emotion regulation and psychological resilience

Integrating allostatic load, the broaden-and-build theory, and COR/protective-factors accounts suggests a sequential process. Stress may first deplete emotion-regulation (ER) capacity; effective strategies such as cognitive reappraisal and mindful awareness may then cultivate positive affect and meaning-making that support the building and maintenance of resilience-related resources and perceived resilience capacity (PR) over time. In this framework, PR is modeled as a downstream resilience capacity that may be shaped by proximal regulatory processes; in turn, stronger PR may dampen aggressive responding—yielding a “regulation-first, resource-next” logic. Evidence supports the proximal segment of this chain: in a three-wave study, earlier emotional competence predicted later resilience, whereas the reverse path was non-significant (Zheng et al., 2021); sequential-mediation models with university samples have shown that emotional abilities promote adaptation via lower stress and higher resilience (Kartol et al., 2024; Gey et al., 2025). Mechanistically, reappraisal appears to build resilience through enhanced mindful attention and acceptance (Zarotti et al., 2020), and in adventure/mountain-training cohorts, emotional abilities covary positively with resilience, extending external validity to athletic contexts (Gavín-Chocano et al., 2023).

On the distal segment, resilience is reliably and inversely linked to aggression and has frequently been modeled as a mediator linking stress (or negative affect) to aggressive behavior. Undergraduate and adolescent studies have traced clear paths from emotional maltreatment through reduced resilience (and self-esteem) to aggression (Chen et al., 2022; Cao and Ma, 2025); among nursing majors, aggression partially mediates the association between resilience and interpersonal difficulties (Lee et al., 2022). Longitudinal and intervention work closes the loop: mindfulness has reduced subsequent aggression partly via gains in resilience, and school-based emotional-competence curricula have produced lasting reductions in physical/verbal aggression through decreases in negative affect (Chen et al., 2024; Castillo-Gualda et al., 2018). However, a training-anchored test that positions training-related perceived stress as the chain’s starting point and evaluates the ER → PR sequence in sport-major undergraduates remains scarce. Based on this, Hypothesis 4 is proposed: Emotion regulation and psychological resilience serially mediate the relationship between training-related perceived stress and aggressive behavior.

## 2 Methods

### 2.1 Participants

Undergraduate sport-major students enrolled at Guangzhou Xinhua University constituted the target population. A classroom-based cluster convenience sampling approach was used. Between October 20 and October 30, 2025, trained research staff visited classes according to the course schedule and invited students to complete an online questionnaire by scanning a QR code at the end of the class period. The survey platform time-stamped each submission (start/end time) and automatically generated anonymized IDs in order of access. Of the 750 questionnaires distributed, 39 were excluded *a priori* based on data-quality criteria (completion time <3 min or >20% missing responses), yielding a final analytic sample of *N* = 711 (valid response rate = 94.8%). The sample comprised men (*n* = 638, 89.7%) and women (*n* = 73, 10.3%). Age distribution was 18 years (*n* = 141, 19.8%), 19 years (*n* = 162, 22.8%), 20 years (*n* = 167, 23.5%), and 21–25 years (*n* = 241, 33.9%). Academic year was freshman (*n* = 171, 24.1%), sophomore (*n* = 185, 26.0%), junior (*n* = 187, 26.3%), and senior (*n* = 168, 23.6%). All measures were administered once, anonymously, under a uniform script. To ensure that items on stress, emotion, and behavior referred specifically to the training ecology, training-related anchoring statements were added to the instructions without altering any item content; details of the anchoring language and scoring appear in the Measures section.

The study protocol was approved by the Biological and Medical Ethics Committee of Guangzhou Xinhua University (IRB No. 2025L003) and adhered to the principles of the Declaration of Helsinki. Participants provided electronic informed consent prior to participation. Data were collected anonymously and used solely for academic research and educational improvement; no information was disclosed to third parties without permission.

### 2.2 Measures

All measures were administered via an anonymous online questionnaire. To align the measurement frame with the study focus, training-anchored instructions were appended to each instrument, directing participants to respond with reference to the past month in training/courses, team management, and competition scheduling; item wording was not altered. To reduce respondent burden and harmonize formats across instruments, we administered all measures using five response categories coded from 1 to 5 at the time of data collection (with each scale’s original semantic anchors and reverse-keying rules preserved as closely as possible). Accordingly, the focal variables should be interpreted as training-anchored self-reports (perceived stress, strategy tendencies, resilience capacity, and aggression propensity) rather than objective training-load indices or observed behavioral incident frequencies.

#### 2.2.1 Chinese perceived stress scale (PTS)

The Chinese Perceived Stress Scale-10 (CPSS-10) (Cohen et al., 1983; Lu et al., 2017) was used to assess undergraduates’ perceived uncontrollability, unpredictability, and overload during the past month. In the present study, CPSS-10 scores operationalized training-related perceived stress (PTS), defined as the participant’s subjective appraisal of stress within the training ecology (training/courses, team management, and competition scheduling) rather than objective training load. To ensure that responses specifically referenced this ecology while keeping item content intact, the following training-anchoring instruction was used: “Please respond with reference to experiences in the past month related to training/courses, team management, and competition scheduling.” The CPSS-10 derives from the original PSS-14 and is commonly modeled with a first-order correlated two-factor structure: helplessness/negative affect (items 1, 2, 3, 6, 9, and 10) and self-efficacy/positivity (items 4, 5, 7, and 8; positively worded items are reverse-keyed). In a Chinese university sample (*N* = 1,096), Lu et al. (2017) reported *α* = 0.85 for the total scale (subscales *α* = 0.86/0.83), a two-week test–retest reliability of *r* = 0.70, and moderate correlations with PHQ-9 (*r* = 0.57) and GAD-7 (*r* = 0.59); CFA supported the two-factor model (GFI = 0.940, NFI = 0.925, CFI = 0.939, RMSEA = 0.049, RMR = 0.039), whereas a one-factor solution fit poorly (CFI = 0.628, RMSEA = 0.122), indicating satisfactory adaptation in Chinese contexts. Response format administration. The original PSS response set comprises five frequency options typically coded 0–4. In this study, we administered the same five-category response structure but coded responses from 1 to 5 for cross-measure format harmonization, while retaining the original semantic anchors and reverse-scoring rules (i.e., this is a coding convention applied at administration, not a *post hoc* score “conversion”). Higher scores indicate higher training-related perceived stress. In the present sample, internal consistency was good for the two factors (*α* = 0.888 for helplessness/negative affect; *α* = 0.824 for self-efficacy/positivity), and CFA indicated excellent fit (*χ*^2^/df = 1.176, RMSEA = 0.016, CFI = 0.998, TLI = 0.998), supporting the scale’s structural validity under training-anchored instructions and its use as the independent variable in subsequent mediation analyses.

#### 2.2.2 Emotion regulation questionnaire (ER)

The Emotion Regulation Questionnaire (ERQ-10) (Gross and John, 2003; Zhang and Bian, 2020) was used to assess sport-major undergraduates’ tendencies in emotion-regulation strategy use. The ERQ-10 is grounded in a first-order correlated two-factor model, with 10 items loading on Cognitive Reappraisal (items 1, 3, 5, 7, 8, and 10) and Expressive Suppression (items 2, 4, 6, and 9). Prior work has shown acceptable internal consistencies (reappraisal *α* = 0.75–0.82; suppression *α* = 0.68–0.76) and, in Chinese undergraduate samples, stable psychometrics (reappraisal/suppression *α* = 0.831/0.778; Satorra–Bentler *χ*^2^/df = 5.95, CFI = 0.934, TLI = 0.929, RMSEA = 0.056, SRMR = 0.038). To align responses with the training context without altering item wording, the training-anchoring instruction was: “Please respond with reference to experiences in the past month related to training/courses, team management, and competition scheduling.” Response format administration. Although the original ERQ uses a 7-point agreement scale, in this study the ERQ-10 was administered using a 5-point Likert agreement format (1–5) to harmonize response formats across instruments and reduce respondent burden; item wording and reverse-keying logic were retained. Construction of a single ER indicator (methodological rationale). In the hypothesized serial mediation framework, ER was conceptualized as a proximal, modifiable regulation resource linking stress to downstream adaptation (resilience) and aggression propensity, rather than as a test of differential effects of specific strategies. To maintain model parsimony and align with the single-mediator role of ER in the PROCESS mediation specifications, we constructed a training-anchored composite ER indicator reflecting an overall adaptive regulation tendency/profile—more endorsement of reappraisal and less endorsement of suppression. Specifically, suppression items (2, 4, 6, and 9) were reverse-keyed and an item-mean composite was computed across all 10 items, with higher scores indicating more reappraisal and less suppression. This composite should be interpreted as a self-reported, training-anchored regulation tendency (not a direct behavioral performance index), and strategy-specific analyses (reappraisal *vs.* suppression modeled separately) remain an important direction for future work. To support the interpretability of this single indicator, we additionally tested a second-order CFA in which a higher-order latent “emotion-regulation tendency” was indicated by the first-order reappraisal and suppression factors; model fit was good (*χ*^2^/df = 1.260, RMSEA = 0.019, CFI = 0.997, TLI = 0.996). Internal consistency in the present sample was strong for both strategies (reappraisal *α* = 0.873; suppression *α* = 0.827), supporting the reliability of the ERQ-10 under the training-anchored, 5-point administration and the use of the composite ER indicator in subsequent mediation analyses.

#### 2.2.3 Brief resilience scale

The Brief Resilience Scale (BRS-6) (Smith et al., 2008; Fung, 2020) was used to operationalize Psychological Resilience (PR) by assessing sport-major undergraduates’ perceived capacity to “bounce back” after stress or setbacks. Consistent with the training-anchored past-month instruction, PR is interpreted here as a context-sensitive resilience capacity under current training conditions rather than a purely trait-like disposition. To align measurement with the study context without altering item wording or scoring, the training-anchoring instruction was: “Please respond with reference to experiences in the past month in training/courses, team management, and competition scheduling.” The BRS-6 is typically modeled as a first-order single-factor (“bounce-back”) scale and comprises six items rated on a 1–5 agreement scale; items 2, 4, and 6 are reverse-keyed. After reverse-keying the relevant items, responses were averaged to yield the PR index, with higher values indicating stronger resilience. Prior work has shown acceptable reliability and evidence of unidimensional structure, and in a Chinese sample (*n* = 511), Fung reported *α* = 0.71 and good confirmatory fit with method-effect allowances. In the present sample, internal consistency was strong (*α* = 0.888), and CFA indicated good fit for the single-factor model (*χ*^2^/df = 1.476, RMSEA = 0.026, CFI = 0.998, TLI = 0.997), supporting the scale’s structural stability under training-anchored administration and its use as the second mediator in subsequent analyses.

#### 2.2.4 Brief aggression questionnaire

A psychometrically equivalent bilingual (Chinese–English) version of the Brief Aggression Questionnaire (BAQ-12) (Webster et al., 2014) was used to assess self-reported aggression propensity among sport-major undergraduates. Without altering item wording or scoring logic, the training-anchoring instruction was: “Please respond with reference to your typical behavior in the past month in training, team interactions, and competition/selection settings; when items are phrased generally, answer according to these training-related contexts.” Accordingly, AB in this study should be interpreted as training-anchored self-reported aggressive tendencies, rather than objectively observed aggressive incidents or event frequencies. The BAQ-12 is a short form of the Buss–Perry Aggression Questionnaire and retains four three-item first-order facets—Physical Aggression (items 1–3), Anger (4–6; item 4 reverse-keyed: “I am an even-tempered person”), Verbal Aggression (7–9), and Hostility (10–12). Consistent with our measurement specification, AB was evaluated using a second-order factor model, in which the four first-order facets load onto a higher-order general aggression factor. For the mediation analyses, we used the BAQ-12 total score (item-mean across all 12 items) as the observed AB indicator, consistent with interpreting the total as a general aggression propensity index. Response format administration. Although the original BAQ-12 uses a 7-point agreement scale, in this study it was administered using a 5-point Likert format (1–5) for format harmonization; item wording and reverse-keying rules were retained. In the present sample, subscale internal consistencies were acceptable to good (*α* = 0.864 for Physical Aggression; *α* = 0.848 for Anger; *α* = 0.838 for Verbal Aggression; *α* = 0.885 for Hostility), and CFA indicated acceptable fit for the specified structure (*χ*^2^/df = 2.972, RMSEA = 0.053, CFI = 0.979, TLI = 0.972), supporting the use of the BAQ-12 total score as the outcome indicator in subsequent analyses.

### 2.3 Statistical analysis

All analyses were conducted in SPSS 27.0, Amos 26, and PROCESS v4.2; all tests were two-tailed with the significance threshold set at *α* = 0.05. Confirmatory factor analyses (CFAs) were first performed in Amos to evaluate measurement-model fit and ensure structural adequacy, followed by Harman’s single-factor test (unrotated principal components analysis) as a preliminary appraisal of common-method variance. After recoding reverse-keyed items and computing item-mean composite scores where applicable, descriptive statistics (M, SD) and Pearson zero-order correlations (r) were computed. Mediation analyses were implemented using the PROCESS macro (Model 6), which simultaneously estimates the two specific indirect effects (PTS → ER → AB and PTS → PR → AB) and the serial indirect effect (PTS → ER → PR → AB), along with the total effect (c) and direct effect (c′). To maintain parsimony and focus on the proposed mechanism, no demographic covariates (e.g., sex and age) were included in the primary models; future studies should test covariate-adjusted and moderated mediation specifications. Indirect effects were estimated via nonparametric bootstrapping with 5,000 resamples to obtain percentile-based 95% confidence intervals; intervals not containing zero were interpreted as statistically significant.

## 3 Results

### 3.1 Assessment of common method bias

Because all measures were obtained from the same cohort of respondents in a single survey session, potential common method variance was examined using Harman’s single-factor test (unrotated principal components analysis) applied to all items. Nine components with eigenvalues greater than 1 were extracted; the first unrotated component had an eigenvalue of 7.84 and accounted for 20.62% of the total variance, which is below the commonly used 40% threshold. No single dominant factor emerged, suggesting that common method variance was unlikely to be a major concern in this dataset.

### 3.2 Descriptive statistics and correlation analysis

As shown in Table 2, the four focal variables exhibited midrange means and dispersion on the 1–5 scale (reverse-scored items were recoded): Perceived Training Stress (PTS), M = 3.01, SD = 0.63; Emotion Regulation (ER), M = 2.98, SD = 0.62; Psychological Resilience (PR), M = 2.94, SD = 0.71; and Aggressive Behavior (AB), M = 3.00, SD = 0.52. Distributions approximated normality, with no substantial departures indicated by skewness and kurtosis. Zero-order correlations were as follows: PTS was positively associated with AB (*r* = 0.220, *p* < 0.001) and negatively associated with ER (*r* = −0.165, *p* < 0.001) and PR (*r* = −0.254, *p* < 0.001); ER was positively associated with PR (*r* = 0.255, *p* < 0.001), and both ER and PR were negatively associated with AB (ER–AB: *r* = −0.140; PR–AB: *r* = −0.191; both *p* < 0.001). Overall, these associations were small to small-to-moderate in magnitude (*r* ≈ 0.140–0.255) and supported proceeding with mediation and serial mediation analyses.

**Table 2:** Mean, standard deviations, and correlations among variables.

Variable	M	SD	1	2	3	4
Perceived training stress (PTS)	3.01	0.63	1			
Emotion regulation (ER)	2.98	0.62	−0.165	1		
Psychological resilience (PR)	2.94	0.71	−0.254	0.255	1	
Aggressive behavior (AB)	3.00	0.52	0.220	−0.140	−0.191	1

*N* = 711. Values are Pearson correlations. Two-tailed tests; all correlations *p* < 0.001.

### 3.3 Serial mediation test of emotion regulation and psychological resilience

**Figure 3:**
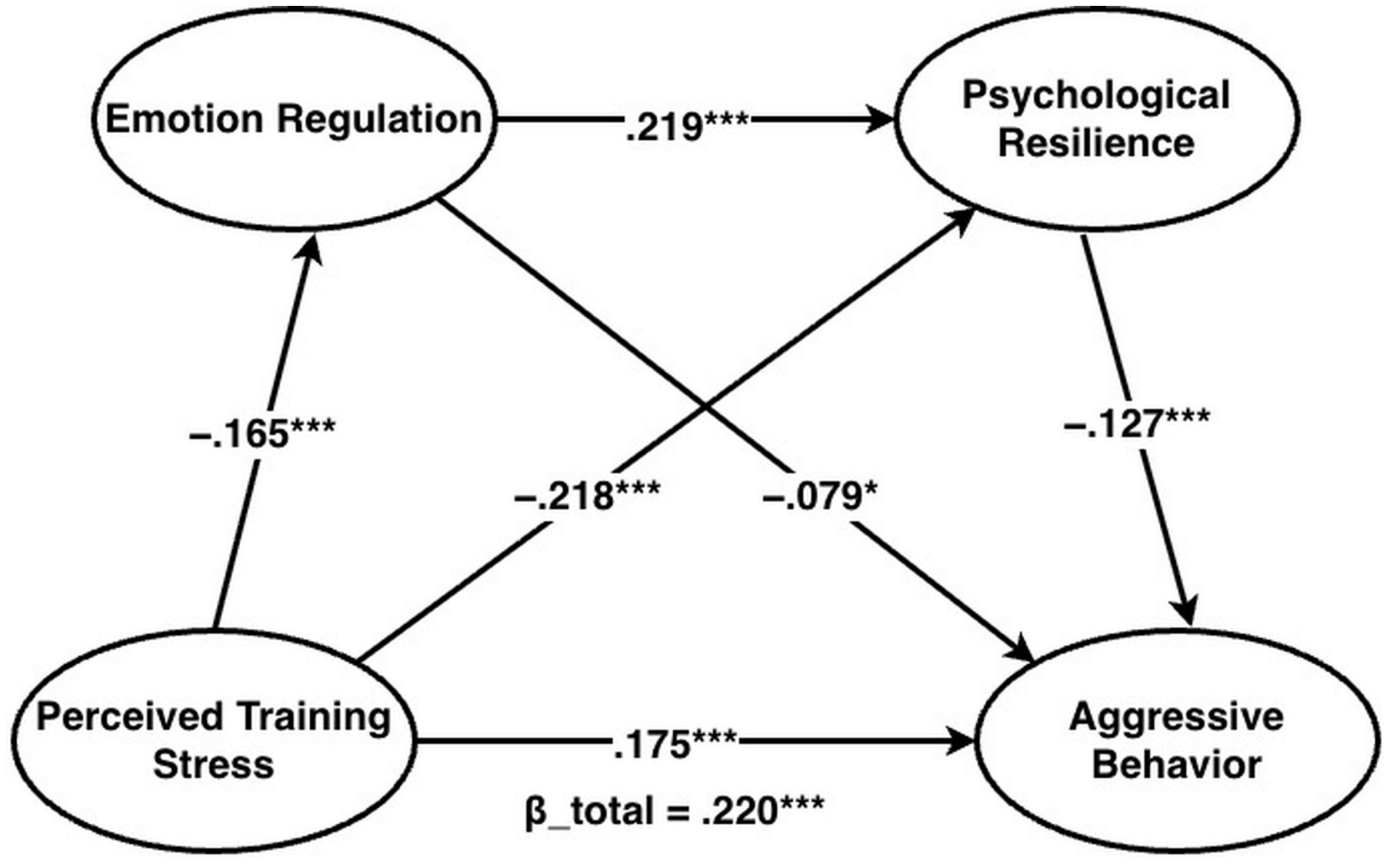
Standardized serial mediation results. Standardized path coefficients (β) from the serial mediation model (PTS → ER → PR → AB) in the present sample (N = 711). β_total denotes the total effect of PTS on AB, and β_direct denotes the direct effect controlling for ER and PR. *p* < 0.05, *p* < 0.01, *p* < 0.001. Unstandardized effects and 95% percentile bootstrap confidence intervals (5,000 resamples) are reported in Table 3. PTS, training-related perceived stress; ER, emotion regulation (higher scores indicate more reappraisal and less suppression); PR, psychological resilience (training-anchored perceived bounce-back capacity); AB, aggressive behavior.

In Amos 26, the overall four-construct measurement model—estimated via a joint CFA of the four instruments (PTS, ER, PR, and AB)—showed good fit (*χ*^2^/df = 1.190, RMSEA = 0.016, CFI = 0.991, TLI = 0.990), supporting the adequacy of the combined measurement model for subsequent analyses. Using PROCESS Model 6 (bootstrap = 5,000; *N* = 711), unstandardized coefficients (B) with 95% confidence intervals (CI) were estimated. PTS was negatively associated with ER (*B* = −0.163, 95% CI [−0.234, −0.091]) and PR (*B* = −0.245, 95% CI [−0.324, −0.166]), and ER positively predicted PR (*B* = 0.249, 95% CI [0.169, 0.329]). With both mediators included, the ER → AB path was negative (*B* = −0.067, 95% CI [−0.129, −0.004]) and the PR → AB path was negative (*B* = −0.094, 95% CI [−0.150, −0.038]); the direct PTS → AB effect remained significant (c′ = 0.145, 95% CI [0.084, 0.207]). The model explained 2.7% of the variance in ER (*R^2^* = 0.027), 11.1% in PR (*R^2^* = 0.111), and 7.4% in AB (*R^2^* = 0.074). Figure 3 presents the standardized solution (*β*_total = 0.220; *β*_direct = 0.175).

For indirect effects (Table 3), all three 95% bootstrap confidence intervals excluded zero. The ER-mediated effect was *B* = 0.011 (95% CI [0.001, 0.024]), the PR-mediated effect was *B* = 0.023 (95% CI [0.009, 0.040]), and the serial ER → PR effect was *B* = 0.004 (95% CI [0.001, 0.007]). The total indirect effect was *B* = 0.038 (95% CI [0.020, 0.058]), accounting for approximately 20.6% of the total effect (c = 0.183), while the direct effect (c′ = 0.145) remained significant, indicating partial mediation. Among the indirect effects, the PR pathway was numerically the largest. Overall, the mediation results were consistent with the hypothesized indirect effects. Hypothesis testing summary. Supporting H1, training-related perceived stress (PTS) was positively associated with aggressive behavior (AB) (total effect: *B* = 0.183, 95% CI [0.123, 0.243]; *r* = 0.220, *p* < 0.001). Supporting H2, the indirect effect of PTS on AB through emotion regulation (ER) was significant (*B* = 0.011, 95% CI [0.001, 0.024]). Supporting H3, the indirect effect through psychological resilience (PR) was significant (*B* = 0.023, 95% CI [0.009, 0.040]). Supporting H4, a small but robust serial indirect effect through ER followed by PR was observed (*B* = 0.004, 95% CI [0.001, 0.007]).

**Table 3:** Mediating effect test and effect size.

Path	Unstandardized effect (B)	Proportion of total effect	Completely standardized effect	95% CI	
				LL	UL
PTS → ER → AB	0.011	5.9%	0.013	0.001	0.024
PTS → PR → AB	0.023	12.6%	0.028	0.009	0.040
PTS → ER → PR → AB	0.004	2.1%	0.005	0.001	0.007
Total indirect	0.038	20.6%	0.046	0.020	0.058
Direct effect (c′)	0.145	—	0.176	0.084	0.207
Total effect (c)	0.183	—	0.222	0.123	0.243

Effects are unstandardized (B). Indirect-effect CIs are 95% percentile bootstrap confidence intervals (5,000 resamples). Completely standardized effects were computed as B × SD (PTS)/SD (AB) using SDs from Table 2. *N* = 711.

## 4 Discussion

Drawing on sport-major undergraduates aged 18–25 at Guangzhou Xinhua University, this study examined a training-anchored serial mediation model linking training-related perceived stress (PTS) to aggressive behavior (AB) through emotion regulation (ER) and psychological resilience (PR). Results indicated that higher PTS was associated with higher AB and that ER and PR accounted for significant indirect pathways, including a small serial pathway consistent with a “regulation-first, resource-next” sequence. These findings extend stress–aggression accounts to a collegiate training ecology and highlight modifiable regulation- and resilience-related targets for prevention, while inferences about temporal ordering remain tentative given the cross-sectional, single-site, self-report design. The sections that follow elaborate the interpretation, applied implications, and boundary conditions.

### 4.1 Perceived training stress and aggressive behavior

Training-related perceived stress (PTS) showed a positive association with aggressive behavior (AB) in the training-anchored context. This association remained significant after accounting for emotion regulation (ER) and psychological resilience (PR), indicating partial mediation and leaving room for additional mechanisms beyond ER and PR. Convergent evidence across populations and settings indicates that sleep restriction or insufficiency often co-occurs with elevations in perceived stress and aggression (Demichelis et al., 2022), social stress can influence hostile appraisal and impulse control via HPA-axis and inflammatory pathways (Takahashi et al., 2018), and increases in external stress levels are accompanied by higher reports of partner aggression and domestic violence (Campbell, 2020; Eckhardt and Parrott, 2017). Within the General Aggression Model (GAM) and General Strain Theory (GST), higher stress in training ecologies can be conceptualized as a situational input and strain that elevates physiological arousal and negative affect, strengthens hostile attributions, and weakens normative evaluation, thereby increasing the accessibility of aggressive scripts. When perceived loss of control or unfairness is salient, anger pathways may be more readily activated, yielding a convergent “strain → anger → aggression” linkage across GAM/GST. In high-load training contexts characterized by public evaluation and roster competition, this pathway is both theoretically grounded and empirically plausible.

Evidence from educational settings and the general population aligns with this pattern. Among first-year undergraduates and adolescents, perceived stress is positively related to cyber or campus aggression, and the direct link often remains observable even after including mediators or controlling related psychological factors (Gao et al., 2024; Guo et al., 2023; Wei et al., 2022). In adult community samples, psychological distress (including a stress component) correlates with anger, hostility, and verbal/overall aggression (Chabbouh et al., 2023; Karam et al., 2024). Some studies further suggest a tighter coupling with reactive aggression (Brown et al., 2017); under isolation, the stress–aggression association can persist despite favorable family climate and social support (Gonultas et al., 2025). Intervention work also indicates that reductions in stress are often accompanied by decreases in aggression, implying potential malleability of this linkage (Guan et al., 2024). Taken together, beyond ER and PR pathways, additional mechanisms—such as inadequate sleep/recovery and perceptions of unfairness or loss of control (mapping onto Demichelis et al., 2022 and the “unfairness–anger” branch in GST)—may help explain why the direct effect persisted after accounting for ER and PR. Practically, a GAM-informed focus on situational inputs and a GST-informed buffering approach would emphasize monitoring load–recovery and sleep at high-pressure nodes, improving rule communication and fairness-focused feedback, and embedding conflict-management and emotion-skills routines around high-stress periods to potentially weaken the PTS–AB association.

### 4.2 Independent mediation effects of emotion regulation and psychological resilience

The indirect-effect decomposition indicated that both single-mediator paths via emotion regulation (ER) and psychological resilience (PR) were statistically significant, with the PR pathway accounting for a larger share of the total association; overall indirect effects were modest in magnitude, consistent with partial mediation. Regarding the ER pathway, longitudinal work suggests that peer victimization and stressful life events prospectively predict increases in emotion dysregulation, which in turn forecasts later increases in aggression (Herts et al., 2012). Experimental studies provide complementary evidence that acute stress can weaken the down-regulatory impact of cognitive reappraisal on anger and fear and may reduce reappraisal effectiveness in fear-conditioning paradigms (Raio et al., 2013; Zhan et al., 2017). This pattern aligns with allostatic-load accounts in which stress taxes prefrontal–limbic control resources and with the GAM/GST appraisal–self-control route: when regulatory capacity is constrained, hostile attributions may become more accessible and inhibitory thresholds may rise. Reviews and meta-analyses also indicate that emotion-regulation difficulties (e.g., greater reliance on suppression, rumination, and impulsive responding) are associated with higher aggression, whereas mindfulness-based approaches can reduce anger and aggression to small-to-moderate degrees, partly via improved regulation processes (Gillions et al., 2019; Roberton et al., 2012). Strategy-level evidence converges: higher mindfulness is indirectly related to lower aggression through reductions in rumination and expressive suppression (Kim et al., 2022); moreover, ER has been shown to mediate associations between other psychosocial predictors (e.g., self-esteem) and aggression (Garofalo et al., 2016). Within sport-training ecologies, high load and uncertainty may tax regulatory resources, making reappraisal more difficult to deploy while suppression and rumination are more likely to dominate; in this context, the statistically significant ER-mediated effect observed here was small.

In parallel, the PR pathway reflected a comparatively stronger resource-based route. In high-pressure occupational groups, job stress has been linked to anger/aggression partly through reduced resilience (Doyle et al., 2021), consistent with Conservation of Resources theory that stress signals threats to and depletion of key resources. Structural models in adolescent samples likewise indicate that negative life events can be associated with higher aggression via declines in resilience (often following reductions in self-esteem) (Yang et al., 2023). In high-risk clinical populations, links between childhood adversity and aggression are reported to operate largely through resilience (Dambacher et al., 2022), and in stressful healthcare contexts resilience has been reported to mediate or moderate associations between anger and aggression (Pachi et al., 2023), suggesting that stronger “bounce-back” capacity may attenuate stress-related hostility and impulse expression. These findings align with protective-factors models of resilience, where resilience—via goal persistence, problem solving, and mobilization of support—may interrupt stress-to-aggression pathways. Given the coefficient pattern in the present study (a larger PR-mediated effect than the ER-mediated effect), PR may function as a comparatively broader, resource-based pathway within training–competition ecologies and may be particularly relevant for longer-term adaptation. Notably, the PR-mediated pathway accounted for a larger proportion of the total effect than the ER-mediated pathway (Table 3; 12.6% *vs.* 5.9%). Statistically, this pattern is consistent with the stronger constituent links involving PR in the estimated model, yielding a larger indirect product. Conceptually, PR captures a broader resilience-related resource capacity (e.g., recovery, persistence, coping, and support mobilization) that may be more directly tied to behavioral self-control and externalizing risk under sustained training strain than an overall composite tendency of regulation strategies. This may help explain why resource depletion reflected by PR contributed a comparatively larger share of the stress–aggression association in the present training-anchored context.

### 4.3 Serial mediation of emotion regulation and psychological resilience

Beyond the two single-mediator paths, a statistically significant but small serial indirect pathway through ER followed by PR was observed, accounting for a small fraction of the total association (approximately 2.1%); together, the indirect pathways accounted for about one-fifth of the total effect. At the beginning of the proposed chain, multiple lines of evidence indicate that stronger cognitive reappraisal/emotion-regulation capacity is positively associated with resilience across cultures, age groups, and instruments (Kökçam et al., 2022; Li and Quan, 2025; Mestre et al., 2017; Sarrionandia et al., 2018; Stover et al., 2024), suggesting that higher-quality emotional processing may contribute to resource building through positive affect and meaning construction, consistent with Broaden-and-Build theory. From an allostatic-load perspective, stress may first deplete regulatory resources; thus, a “regulate first, resource next” sequence has plausible psychophysiological grounding. Converging evidence also positions emotional-processing channels as important links between stress/adversity and problem behaviors (Ong and Thompson, 2019; Zhou et al., 2024), which is consistent with the serial logic tested here.

At the distal end of the chain, adolescent and collegiate samples consistently document an inverse association between resilience and aggression and show that resilience can mediate—or serve as a key mediator of—the link between stress (or adverse life events) and aggression (Arslan, 2016; Yang et al., 2023; Zhang et al., 2023), indicating that stronger recovery capacity may dampen downstream externalizing responses. This pattern aligns with protective-factors models of resilience and Conservation of Resources theory: stress represents a threat to resources, whereas resilience may counteract behavioral consequences via goal persistence, problem solving, and mobilization of support. In applied terms, the coefficient pattern is consistent with a stepped focus in training settings: reducing salient stressors, strengthening near-term regulation skills, and supporting longer-term resilience resources. Importantly, temporal ordering cannot be established from cross-sectional data; therefore, the serial interpretation should be viewed as a statistical pattern consistent with the proposed sequence that requires longitudinal confirmation. Taken together, external evidence and the present estimates were consistent with the proposed serial mediation pattern, while longitudinal confirmation remains necessary.

### 4.4 Limitations and future directions

This study has several methodological and sampling constraints that warrant caution in interpretation. The sample consisted of sport-major undergraduates from a single university, with a relatively homogeneous regional context and training culture; the sex ratio was imbalanced and no sex-stratified analyses were conducted, limiting generalizability. Although age was recorded, sex and age were not modeled as covariates in the primary analyses; given the pronounced sex imbalance (and the relatively small female subsample), sex-stratified or sex-specific model comparisons were not pursued. Future research should recruit more balanced samples and examine sex- and age-related differences using covariate-adjusted and measurement-invariance approaches. In addition, measurement validation (CFA) and hypothesis testing were conducted in the same sample; split-sample or independent-sample cross-validation would further reduce overfitting concerns and strengthen generalizability. The design was cross-sectional and relied on a single administration of self-report instruments; although Harman’s single-factor test suggested that common-method variance was not dominant, same-source measurement and social-desirability effects cannot be ruled out. The use of training-related anchoring and scale rescaling improved contextual alignment but may have introduced method effects and reduced comparability with studies using the original response formats. The model did not include several plausible mediators or moderators—such as sleep and recovery, perceived unfairness and loss of control, coach–athlete relationship, team climate, and objective training load. Classroom cluster administration may also have introduced within-class dependence; multilevel modeling or cluster-robust standard errors were not applied. The serial indirect effect was small, underscoring that statistical significance does not necessarily imply practical significance and that effect estimates may vary across training phases. In addition, collapsing the two ERQ strategies into a single composite may have obscured strategy-specific effects; sensitivity checks using strategy-specific or alternative mediation specifications are therefore advisable.

Future work can advance along three fronts—design, measurement, and intervention. In design, multi-wave longitudinal studies, weekly diaries, or ecological momentary assessment can test the temporal ordering of the “stress → emotion regulation → resilience → aggression” sequence; cross-lagged latent-variable approaches and multilevel models (or cluster-robust standard errors) can help disentangle within-person change from between-person differences. Replication across multiple universities and regions with measurement-invariance testing is encouraged, alongside comparisons across training phases (e.g., early adaptation vs. key in-season windows), sport types, sex subgroups, and age groups. In measurement, incorporating coach/peer ratings, classroom observation, and wearable sensors could yield more objective indicators of training load, heart-rate variability, and sleep; marker variables or latent method factors within a multitrait–multimethod framework could further address common-method concerns. Administering both original and rescaled versions of the instruments in parallel and conducting sensitivity analyses would strengthen comparability. Future studies may also incorporate sport-specific resilience instruments (or sport-adapted versions) alongside generic measures to strengthen domain specificity and facilitate cross-study comparability in sport-training contexts. Mechanistically, expanding candidate mediators (e.g., sleep quality, perceived fairness and control, coach–athlete relationship, team climate) and testing conditional indirect effects and competing models—while probing nonlinearity/thresholds and potential reciprocal paths (e.g., resilience feeding back to regulation)—will refine the framework. On the practice side, stepped randomized controlled trials are warranted: at high-pressure training nodes, first reduce salient stressors and strengthen emotion regulation (e.g., reappraisal scaffolding, attention-breathing practice), then cultivate resilience through positive emotion and meaning-making, with both behavioral and physiological endpoints; preregistration and data sharing will enhance reproducibility and translational value. Given the frequent critique in sport contexts that correlational findings are not sufficiently translated into practice, intervention-focused studies are particularly important for demonstrating feasibility, effectiveness, and implementation value.

## 5 Conclusion and recommendations

Within a measurement framework anchored to training contexts, analyses of the sport-major undergraduate sample indicated that training-related perceived stress was significantly associated with aggressive behavior, and the direct association remained significant after emotion regulation and psychological resilience were included simultaneously. Two independent indirect pathways were observed (via emotion regulation and via psychological resilience), along with a statistically significant serial indirect pathway (stress → emotion regulation → psychological resilience → aggression). Although the serial indirect effect was small, the combined indirect effects accounted for approximately one-fifth of the total association, consistent with the notion that a more immediate regulatory process and a broader resilience-related resource capacity may operate in tandem within a training ecology. Taken together, the findings are consistent with a testable “stress–regulation–resilience–behavior” framework; given the cross-sectional, single-site, self-report design, the results should be interpreted as associations, and broader generalization and temporal ordering require further verification.

Following a “reduce stress → regulate → build” sequence, practical efforts may be structured as follows. At high-pressure junctures—pre-competition weeks, dense training blocks, and selection periods—programs can consider integrated management of training load, sleep, and recovery, alongside greater process transparency to reduce perceived uncontrollability and unfairness. Brief, training-embedded micro-skills (e.g., cognitive reappraisal, self-distancing, and breathing-attention drills) may be incorporated into daily training and pre-competition routines to support rapid de-arousal in context. Psychological resilience may be cultivated more systematically through goal-tiering, peer support, constructive after-action reviews, and resource linkage, with additional attention to students in peak-stress windows and those showing weaker adaptation. Brief training-anchored monitoring that integrates coach/peer inputs with behavioral records may also support tiered early-warning and confidential referral when needed. At the organizational level, a “small pilot → outcome evaluation → staged rollout” approach may help ensure that any intervention package is feasible, sustainable, and scalable.

## Data Availability

The original contributions presented in the study are included in the article/supplementary material, further inquiries can be directed to the corresponding author.
